# Systemic Therapies for Desmoid Tumors: A Review of Past, Present, and Future Treatments

**DOI:** 10.3390/cancers18101521

**Published:** 2026-05-09

**Authors:** Skylar L. Nahi, Amanda M. Dann

**Affiliations:** Division of Surgical Oncology, Department of Surgery, The University of Texas Southwestern Medical Center, Dallas, TX 75390, USA; skylar.nahi@utsouthwestern.edu

**Keywords:** desmoid tumor, aggressive fibromatosis tamoxifen, NSAID, methotrexate, doxorubicin, sorafenib, γ-secretase inhibitors, nirogacestat

## Abstract

Desmoid tumors (DTs) are rare, fibroblastic neoplasms characterized by locally aggressive behavior, unpredictable clinical trajectories, and a substantial impact on patient quality of life despite minimal metastatic potential. Although the underlying biology of DTs remains incompletely defined, associations with prior trauma, hormonal exposure, and aberrant Wnt/β-catenin signaling—including somatic CTNNB1 mutations and germline APC alterations seen in Familial Adenomatous Polyposis—have informed both historical and contemporary therapeutic approaches. Management strategies have evolved from surgery-dominant paradigms toward individualized, multimodal treatment algorithms emphasizing systemic medical therapy, as reflected in current NCCN and Desmoid Tumor Working Group recommendations. This review focuses on the medical management of DTs, tracing the evolution from earlier noncytotoxic therapies, including antiestrogen agents such as tamoxifen, to modern systemic options supported by prospective and randomized data. We summarize available evidence for four principal classes of medical therapy: nonsteroidal anti-inflammatory drugs, cytotoxic chemotherapy (with particular emphasis on anthracycline-based regimens), tyrosine kinase inhibitors—most notably sorafenib—and the emerging class of γ-secretase inhibitors. Recent phase III data supporting the efficacy of nirogacestat highlight a shift toward mechanism-based, targeted treatment with demonstrable benefits in progression-free survival, symptom control, and patient-reported outcomes. Collectively, these advances underscore a maturing therapeutic landscape in which systemic therapy plays a central role in disease control, symptom palliation, and preservation of function for patients with advanced desmoid tumors.

## Simple Summary

Desmoid tumors are rare soft tissue tumors that do not usually spread to other organs, but can grow aggressively where they arise and cause major pain, functional impairment, and reduced quality of life. Treatment has shifted away from older hormone-based approaches, which showed limited and inconsistent benefit, toward more effective systemic therapies such as anthracycline-based chemotherapy, tyrosine kinase inhibitors, and newer γ-secretase inhibitors. Among recent advances, nirogacestat has emerged as an important targeted therapy with strong evidence for improving tumor control, symptoms, and patient-reported quality of life. This review summarizes how systemic treatment for desmoid tumors has evolved and highlights promising directions for more personalized, biology-based care.

## 1. Introduction

Desmoid tumors (DT) are rare fibroblastic neoplasms arising from connective tissue that can develop throughout the body and exhibit variable clinical behavior. While our understanding of the etiology of these tumors remains incomplete, there is an association of these tumors with a history of trauma and suggestion of a potential hormonal role in their development. Somatic mutations in β-catenin (CTNNB1), adenomatous polyposis coli (APC) gene mutations and germline APC mutations in Familial Adenomatous Polyposis (FAP) syndrome are also associated with DTs [1,2,3].

Incidence is documented at approximately 2–4 persons per million per year, and can manifest with a significant clinical burden and negatively impact quality of life [1]. Although metastatic potential is minimal and mortality is low, patients can suffer the morbidity of locally aggressive tumors including: acute on chronic pain, swelling, and subsequent mass effect, which, based on location, can result in decreased range of motion, bowel obstruction, compromise of visceral organs, and more. The natural history of the disease evolution and progression is variable, and the National Comprehensive Cancer Network (NCCN) clinical practice guidelines and the Desmoid Tumor Working Group (DTWG) have proposed management guidelines that are nuanced and structured around multidisciplinary care [4,5,6]. Given the potential for prolonged stability, or even spontaneous regression, current guidelines support active surveillance as the first step in management for many patients with DT. However, for the significant portion of patients who progress or develop symptoms, multiple therapeutic options exist. Interventions include surgery, radiotherapy, other locoregional therapy (cryoablation, high-intensity-focused ultrasound, radiofrequency ablation), and notably, systemic medical therapies. This review will discuss the medical therapies for desmoid tumor, highlighting more historic treatments including tamoxifen with less of a presence in the current DT treatment landscape, and an overview of currently employed therapies supported by the most recent data.

## 2. Methods/Literature Search Strategy

A narrative review with a structured literature search was performed. The review was designed to provide a comprehensive, clinically oriented overview of the evolution of systemic therapies for desmoid tumors. The search strategy was designed to be broad and reproducible, and evidence synthesis was guided by multidisciplinary clinical expertise to provide a balanced overview of the field. This narrative review was prepared with attention to the SANRA framework for narrative reviews, with emphasis on a clearly defined clinical aim, transparent description of the literature search, appropriate referencing, and balanced synthesis of the available evidence. To reduce the risk of undue selection bias, the review used a broad, predefined multi-database search strategy supplemented by reference cross-checking, and evidence was incorporated on the basis of clinical relevance, historical importance, and practice-changing impact.

The literature search was conducted using PubMed/MEDLINE, EMBASE, and ClinicalTrials.gov from inception through January 2026. Search terms included “desmoid tumor,” “desmoid fibromatosis,” “aggressive fibromatosis,” “CTNNB1,” “systemic therapy,” “chemotherapy,” “tyrosine kinase inhibitor,” “gamma-secretase inhibitor,” “nirogacestat,” “sorafenib,” “doxorubicin,” “methotrexate,” “tamoxifen,” and “NSAID,” used in various Boolean combinations. Inclusion criteria were: (1) clinical studies, trials, or reviews reporting systemic medical therapies for desmoid tumors in adult or pediatric populations; and (2) studies reporting at least one of the following outcomes: overall response rate, progression-free survival, overall survival, or toxicity profile. Case reports and studies with fewer than five patients were excluded. Reference lists of selected articles were manually reviewed to identify additional relevant studies. Articles were selected for relevance to systemic medical treatment of desmoid tumors, with emphasis on prospective trials, influential retrospective series, clinical practice-informing studies, and key historical reports that shaped treatment development. Outcomes of interest included response, disease control, progression-free survival, toxicity, symptom burden, and patient-reported benefit where available.

## 3. Body and Discussion

In the history of desmoid tumor treatment, patterns in prevalence have revealed possible risk factors, and guided early interventions and therapies. For example, patterns of prevalence and incidence revealed a possible relationship between DTs and hormone exposure. Epidemiologic studies have consistently noted a female preponderance and disproportionate number of childbearing age women afflicted with desmoid tumors [7,8,9]. For example, a representative study in Denmark in 2022 of 179 patients with desmoid tumors demonstrated 76% were female with a median patient age of 38 [3]. Furthermore, DT incidence increases during pregnancy, with oral contraceptive use, and in the setting of recent pregnancy [1,2,7,10]. In addition, spontaneous regression of tumor burden has been observed during menopause, or after tamoxifen treatment [9]. As such, an observational theory was developed regarding tumor development: estrogen may influence tumor growth and spontaneous regression.

In the setting of this observation and proposed tumor progression mechanism, desmoid tumor has previously been treated with the selective estrogen receptor modulating (SERM) therapy, tamoxifen. However, an overview of the most recent data does not demonstrate a discrete cause–effect relationship between tamoxifen, or tamoxifen and sulindac, on disease control or tumor regression [Table 1]. Pertinent studies in this space include a systematic review from 2011, which identified 41 studies over approximately 30 years, including 168 patients with DT treated with antiestrogen agents, alone or in combination with nonsteroidal anti-inflammatory drugs. The overall response rate was reported as 51% [10]. Importantly, response was not found to correlate with estrogen receptor status of the tumor. In the setting of a tumor with a natural regressing and remitting phenotype, these data were not consistent with a durable and causative relationship between estrogen exposure/chemically induced absence, and tumor regression [10].

Clinical trials have attempted to investigate if the relationship between hormone fluctuations and DT incidence or progression can be targeted with medical therapy. Specifically, clinical trials evaluating tamoxifen with and without a NSAID have demonstrated safety and an acceptable profile of adverse effects; however, there is insufficient data to conclude rates of regression are reliably above the spontaneous regression rate for DTs. A Children’s Oncology Group phase II trial from 2013 enrolled 59 patients to evaluate safety and efficacy of tamoxifen and sulindac for DT. While serious side effects were limited, with ovarian cysts being the most common, the two year progression-free survival rate was not found to be significantly decreased from the natural history of untreated disease (approximately 36%) [13]. These data are consistent with the consensus in the literature; as such, this noncytotoxic medical therapy regimen is no longer standard of care at sarcoma centers of excellence.

Today, studies and clinical trials have focused efforts on promising new medical therapies that can be organized into four key classes: First, there remains a role for nonsteroidal anti-inflammatory drugs (NSAIDs), which was previously dominated by sulindac and has been expanded to include indomethacin and celecoxib. Second, a cornerstone of modern treatment, cytotoxic agents notably of the anthracycline class, currently best exemplified by Doxil (liposomal doxorubicin). Third, within the category of targeted therapies: tyrosine kinase inhibitors (TKIs), most notably, sorafenib. Fourth and lastly, a newer class of specific and targeted therapies, γ-secretase inhibitors, with representative medications nirogacestat and varegacestat (AL102).

### 3.1. NSAIDs

NSAIDs were explored early in the management of desmoid tumors as relatively well-tolerated noncytotoxic agents; later observations of COX-2 overexpression in desmoid tumors provided a biologic rationale supporting their continued study in this disease [14,15]. In the setting of known, well-tolerated, FDA-approved COX-1 and COX-2 blockers, studies were conducted to investigate whether various NSAIDs could influence desmoid tumor regression and recurrence [Table 2]. However, interpretation of these, and subsequently described, medication response rates requires consideration of the underlying natural history of desmoid tumors. Desmoid tumor natural history is recognized to be highly variable and commonly includes prolonged stability, spontaneous regression, and delayed regression after an initial period of progression [16,17,18]. In a contemporary prospective, multicenter, observational study, amongst a cohort of 108 patients under active surveillance, 39% demonstrated RECIST progression at a median follow-up of 32.3 months, while spontaneous regression was observed initially in 25% and after dimensional progression in an additional 31%, highlighting that meaningful tumor regression may occur in the absence of systemic intervention [19].

Against this background, although NSAIDs in isolation or in combination with other treatments have consistently demonstrated tolerability and a low adverse-effect profile, the reported response rates of agents such as sulindac and meloxicam have not been clearly shown to exceed the baseline rates of spontaneous stabilization or regression expected in the natural course of disease [12,13]. As such, current NCCN guidelines include the use of NSAIDs, for example sulindac, primarily in the role of pain management or as an adjunct to a primary medical therapy [4].

### 3.2. Anti-Metabolites/Cytotoxic Therapies

Methotrexate (MTX), an anti-folate anti-metabolite, is a frequently used chemotherapeutic choice for multiple cancers, and it is also FDA-approved for autoimmune conditions including rheumatoid arthritis. Multiple clinical trials have demonstrated efficacy of a methotrexate-containing regimen to treat recurrent DTs or disease not amenable to local therapies [Table 3]. A phase II trial of 26 patients from 2007 demonstrated measurable response in 8 patients (31%) and 10 patients with stable disease following weekly injection with methotrexate and vinblastine [23]. As with other cytotoxic choices, the efficacy of methotrexate is achieved at the cost of higher rates of adverse effects. In patients treated with MTX for DT, the most common grade 3 and higher adverse effects are neutropenia and transaminitis [14]. More recently, MTX regimens have been replaced by alternative cytotoxic agents. In the systematic review by Tsukamoto et al., a methotrexate containing regimen disease control rate was 71–100%, compared to more currently popular agents such as Doxil (liposomal doxorubicin) with 90–100% [24].

Regarding systemic therapies for desmoid tumor, anthracycline containing regimens have consistently demonstrated efficacy in the last twenty years of published literature and clinical trials [Table 3]. Specifically, Doxil is a pegylated liposomal doxorubicin, and it is a formulation that has overtaken MTX as a primary cytotoxic therapy for the desmoid tumor patient population. Doxorubicin is an anthracycline with multifaceted cytotoxic activity, predominantly linked to topoisomerase II-associated DNA damage, but also mediated through DNA intercalation. More recent data suggest that, during DNA replication, doxorubicin may additionally impair fork progression through a topoisomerase II–independent intercalative mechanism [30]. It has been utilized in the treatment of multiple cancers including: breast, lung, gastric, ovarian, thyroid, non-Hodgkin’s and Hodgkin’s lymphoma, multiple myeloma, sarcoma, and pediatric cancers. Of note, one of the major side effects of doxorubicin is cardiotoxicity, which limits its utilization by patients with poor heart function and can limit tolerable doses and impact desired efficacy. Ultimately, when compared to other drug classes, or other cytotoxic/chemotherapy agents, Doxil demonstrates favorable progression-free survival (PFS) in DT relative to systemic options available at the time of respective publications [26,27,29].

In a 2010 study by Pires de Camargo et al., Doxil demonstrated superior PFS when compared to other notable cytotoxic therapy agents on the market, as well as alternative therapies such as hormonal agents and TKIs [31]. Regarding adverse effects, the study reports two patients, of the 35 who received anthracyclines, who developed cardiotoxicity [31]. Although both patients had improvement in their symptoms with medical management, cardiotoxicity remains one of the key limiting variables in prescribing doxorubicin. Evaluating these data in the context of medical management of DT, recognizing cross-era comparisons with newer targeted agents should be interpreted cautiously; many major cancer centers and sarcoma centers of excellence utilize Doxil as a first-line therapy for patients with progressing DT.

### 3.3. Tyrosine Kinase Inhibitors

TKIs including pazopanib, imatinib and sorafenib have all been employed in desmoid tumors [Table 4]. While pazopanib in some studies has demonstrated lower toxicity than imatinib, among the drug class, sorafenib has the most extensive efficacy data and thus is the preferred agent at present [32,33,34]. Specifically, a 2023 systematic review by Tsukamoto et al. reviewed medical treatment options for progressive desmoid tumors and found that tyrosine kinase inhibitors as a class demonstrated meaningful clinical activity, although cross-study comparisons between individual agents are limited by differences in study design, patient selection, and reported endpoints [24]. More recent real-world comparative data from Noujaim et al. further support the clinical activity of specifically sorafenib and pazopanib in routine practice, while underscoring that TKI selection should be individualized on the basis of efficacy, toxicity profile, and patient-specific considerations [32].

Sorafenib, an oral multitargeted receptor tyrosine kinase inhibitor, has taken a leading role as the primary TKI utilized as a first-line agent for desmoid tumor [24,33]. A leading argument for the mechanism of this medication class in influencing desmoid tumor regression is theorized to be inhibition of PDGFRB kinase activity [35]. In addition to an established and clinically familiar safety profile, sorafenib is distinguished from other TKIs by the strength of its supporting evidence base, particularly the landmark randomized phase III trial by Gounder et al. [34]. With a median follow-up of 27.2 months, the investigators demonstrated a 2-year progression-free survival rate of 81% in the sorafenib group versus 36% in the placebo group [34]. The most commonly reported adverse events were grade 1 and grade 2 events (53% sorafenib v 69% placebo), although 47% of patients developed grade 3 or 4 adverse events compared to 25% in the placebo group. The grade 3 and 4 adverse events were most commonly cutaneous or gastrointestinal, and led to a significantly higher rate of discontinuation of the trial regimen in the sorafenib group than in the placebo group. This landmark study has played a key role in establishing sorafenib as the representative TKI in desmoid tumor management.

### 3.4. γ-Secretase Inhibitors

Finally, a promising new therapy for desmoid tumor, nirogacestat, falls within the final medication class: γ-secretase inhibitors [Table 4]. Just as NSAIDs have been used to target the COX-2 expression pattern of DTs, γ-secretase inhibitors became a therapy of interest after evidence that desmoids highly express Notch1. The Notch signaling pathway has been identified to influentially impact desmoid proliferation, and the γ-secretase enzyme is an integral protein in the signaling pathway, cleaving the Notch intracellular domain and facilitating its nuclear translocation to promote gene transcription [43,44]. γ-secretase inhibitors block Notch signaling and, as such, were theorized to restrain DT growth. In phase I and phase II trials, nirogacestat showed antitumor activity and pain palliation. A 2023 phase III randomized, placebo-controlled trial enrolled patients with progressing desmoids to explore this new medication class. Nirogacestat had a significant progression-free survival benefit over placebo (HR 0.29, *p* < 0.001), and the likelihood of being event-free at 2 years was 76% with nirogacestat and 44% with placebo [41]. The documented adverse events were most commonly (95%) grade 1 or grade 2, although, among women of childbearing potential receiving nirogacestat, 27 of 36 (75%) had adverse events consistent with ovarian dysfunction. Overall, the study robustly demonstrated the medication’s impact on progression-free survival, objective response, pain, disease-specific symptom burden, physical functioning, role functioning, and health-related quality of life, and, subsequently, in November of 2023, the FDA-approved nirogacestat (OGSIVEO) for adult patients with progressing desmoid tumors who require systemic treatment [41]. Further trials are ongoing exploring safety and efficacy of novel γ-secretase inhibitors including the RINGSIDE trial, a phase II/III trial utilizing the agent AL102 in a patient population of adults with progressive desmoid tumor [Table 4].

## 4. Conclusions

In the setting of a rare, heterogeneous soft tissue neoplasm with an often indolent yet potentially morbid clinical course, the medical management of desmoid tumors has undergone a meaningful evolution over the last several decades. Early observational and epidemiologic studies provided foundational insight into potential biologic drivers of disease, including hormonal exposure and aberrant Wnt/β-catenin signaling, which in turn guided the development of initial noncytotoxic therapeutic strategies. However, as prospective data have matured and randomized trials have become feasible in this disease space, the limitations of earlier empiric approaches, particularly antiestrogen-based regimens, have become increasingly apparent.

Contemporary management paradigms now emphasize active surveillance for select patients, followed by a stepwise introduction of systemic therapy when disease progression, symptom burden, or functional compromise necessitate intervention. Within this framework, systemic medical therapy has emerged as a cornerstone of treatment for patients with progressive or symptomatic disease not amenable to local control. Tyrosine kinase inhibitors, most notably sorafenib, have demonstrated durable disease stabilization with a favorable toxicity profile and now represent a commonly employed first-line systemic option at many centers. Further, cytotoxic chemotherapy, particularly anthracycline-based regimens such as pegylated liposomal doxorubicin, remains among the most consistently effective options for disease control, though its use must be balanced against cumulative toxicity and patient-specific comorbidities. Concerns for the potential of cardiotoxicity is typically addressed through baseline cardiac assessment and risk-adapted interval monitoring during treatment, particularly in patients with higher cumulative exposure or underlying cardiovascular risk. During the treatment course, monitoring and surveillance allow for dose adjustment or discontinuation if clinically meaningful cardiac dysfunction emerges.

Perhaps most notably, the development and successful phase III evaluation of γ-secretase inhibition with nirogacestat marks a pivotal advance in the field and the first FDA-approved therapy for management of progressing desmoid tumors. For the first time, a targeted therapy grounded in a defined biologic pathway has demonstrated robust improvements not only in progression-free survival, but also in patient-reported outcomes including pain, physical functioning, and overall quality of life. At the same time, ovarian dysfunction should be recognized as a major treatment-related toxicity of nirogacestat, particularly given the predominance of desmoid tumors in young women. Although ovarian dysfunction may improve after treatment discontinuation in many patients, the degree and timing of recovery may not yet be predictable at the individual-patient level. Accordingly, for patients of reproductive age, this risk warrants explicit pre-treatment counseling regarding menstrual and reproductive effects, consideration of fertility preservation when future childbearing is desired, and early involvement of gynecology or reproductive endocrinology when appropriate. During treatment, monitoring for menstrual irregularity and other symptoms of ovarian dysfunction is warranted, and these risks should be incorporated into shared decision-making alongside the demonstrated benefits of nirogacestat in disease control, symptom burden, and quality of life. These data underscore an important shift toward mechanism-based treatment strategies and highlight the feasibility of conducting high-quality randomized trials in desmoid tumor populations.

## 5. Future Directions

Looking forward, several key areas warrant continued investigation. Regarding targeted therapies, In addition to γ-secretase inhibition, other mechanism-based strategies are emerging, including β-catenin-directed therapies such as tegavivint (BC2059), which disrupts the β-catenin/TBL1-TBLR1 interaction and has shown preclinical activity in desmoid tumor models with early clinical evaluation ongoing [45,46]. Additionally, improved molecular stratification, particularly with respect to CTNNB1 mutation subtype, may refine treatment selection and sequencing, as emerging data suggest differential responses to select medical therapies. Because CTNNB1 encodes β-catenin, a central mediator of the dysregulated Wnt/β-catenin signaling pathway that underlies desmoid tumor biology, mutational subtype of this and related proteins may carry mechanistic as well as clinical relevance. For example, Hamada et al. reported that the CTNNB1 S45F mutation was associated with poor response to meloxicam, and all S45F-mutant tumors in their prospective cohort demonstrated progressive disease [22]. This finding supported the possibility that mutation subtype may have predictive as well as prognostic value in conservative and systemic treatment selection. More broadly, these findings suggest that biologically informed molecular stratification may help guide future personalization of therapy in desmoid tumors, particularly as pathway-directed agents continue to enter clinical practice. In addition, further study is needed to define the optimal timing, duration, and sequencing of systemic agents, especially as newer targeted therapies are integrated into clinical practice. Finally, given the chronic nature of disease for many patients, long-term toxicity, fertility preservation, and survivorship considerations must remain central to therapeutic decision-making.

As the treatment landscape continues to expand, multidisciplinary care anchored in shared decision-making remains essential. Ongoing collaboration between sarcoma centers, cooperative groups, and patient advocacy organizations will be critical to advancing both clinical outcomes and quality of life for patients with desmoid tumors.

## Figures and Tables

**Table 1:** Key reported trials on hormone-based therapy for desmoid tumor/desmoid fibromatosis (DT/DF).

Study/Agent(s)	Year	Intervention/Trial	Pt No.	Overall Survival (OS)	Progression-Free Survival (PFS)	Overall/Objective Response Rate (ORR)	Toxicity (% of Grade 3, 4 Events)
Tamoxifen and sulindac (Hansmann et al.) [11]	2004	High-dose tamoxifen and sulindac as first-line treatment for desmoid (FAP-associated and Sporadic)	25	NR	NR	FAP-associated: 31% ORR (4 PR/CR > 6 mo); Non–FAP-associated: 13% ORR (1 CR)	NR
SERM (tamoxifen, toremifene, raloxifene) and Sulindac (Quast et al.) [12]	2016	High-dose selective estrogen receptor modulators and sulindac for sporadic and FAP-associated desmoid tumors	134	NR	NR	33% (20 CR, 24 PR)	NR

Patient (Pt), Not Reported/Not Reached (NR), Complete Response (CR), Partial Response (PR), Familial Adenomatous Polyposis (FAP).

**Table 2:** Key reported trials on anti-inflammatory therapy for desmoid tumor/desmoid fibromatosis (DT/DF).

Study/Agent	Year	Intervention/Trial	Pt No.	Overall Survival (OS)	Progression-Free Survival (PFS)	Overall/Objective Response Rate (ORR)	Toxicity (% of Grade 3, 4 Events)
Sulindac (Tsukada et al.) [20]	1992	Noncytotoxic drug therapy for intra-abdominal desmoid in patients with FAP	14	NR	NR	57% ORR (1 CR, 7 PR)	NR
Meloxicam (Nishida et al.) [21]	2010	Prospective treatment of extra-abdominal desmoid with meloxicam	22	NR	NR	36% ORR (1 CR, 7 PR)	NR
Meloxicam (Hamada et al.) [22]	2014	Meloxicam for extra-peritoneal sporadic desmoid tumors	33	NR	NR	24% (1 CR, 7 PR)	NR

Patient (Pt), Not Reported/Not Reached (NR), Complete Response (CR), Partial Response (PR), Familial Adenomatous Polyposis (FAP).

**Table 3:** Key reported trials on cytotoxic therapy for desmoid tumor/desmoid fibromatosis (DT/DF).

	Study/Agent	Year	Intervention/Trial	Pt No.	Overall Survival (OS)	Progression-Free Survival (PFS)	Overall/Objective Response Rate (ORR)	Toxicity (% of Grade 3, 4 Events)
Anthracycline -based regimen								
	Doxorubicin + Dacarbazine (Patel et al.) [25]	1993	Doxorubicin + dacarbazine	12	NR	NR	67% ORR (1 CR, 4 PR)	11%
	Doxorubicin + Dacarbazine (Gega et al.) [26]	2006	Doxorubicin, Dacarbazine, + meloxicam for FAP patients with desmoid	7	NR	74 months (range: 32.5 to 107.5 months)	100% ORR (3 CR, 4 PRs)	43%
	Pegylated liposomal doxorubicin (Constantinidou et al.) [27]	2009	Pegylated liposomal doxorubicin for refractory, aggressive fibromatosis	12	NR	NR	36%	8%
Other cytotoxic regimen								
	MTX + Vinblastine (Azzarelli et al.) [28]	2001	Methotrexate + vinblastine/vinorelbine in advanced, aggressive fibromatosis: phase II trial	30	NR	67% (10 year actuarial PFS)	40% ORR (40% PR, 0% CR)	NR
	MTX and Vinblastine (Garbay et al.) [29]	2012	Methotrexate, vinblastine	27	NR	NR	15% RR (4 PR, 14 SD, 9 PD)	NR

Patient (Pt), Not Reported/Not Reached (NR), Response Rate (RR), Complete Response (CR), Partial Response (PR), Stable Disease (SD), Progressive Disease (PD), Familial Adenomatous Polyposis (FAP).

**Table 4:** Key reported trials on targeted therapies for desmoid tumor/desmoid fibromatosis (DT/DF).

	Study/Agent	Year	Intervention/Trial	Pt No.	Overall Survival (OS)	Progression-Free Survival (PFS)	Overall/Objective Response Rate (ORR)	Toxicity (% of Grade 3, 4 Events)
Tyrosine kinase inhibitors	Imatinib (Heinrich et al.) [35]	2006	Imatinib for advanced aggressive fibromatosis: Phase II Clinical Trial	19	NR	36.8% (1 year PFS)	16% ORR (PR 3, CR 0)	NR
	Imatinib: Phase II Multi-Center SARC Trial (Chugh et al.) [36]	2010	Imatinib for aggressive fibromatosis: SARC trial	51	NR	66% (1 year PFS); 58% (3 year PFS)	6% ORR	Neutropenia (*n* = 5), rash (*n* = 5), fatigue (*n* = 5)
	Imatinib Mesylate: French Sarcoma Group (Penel et al.) [37]	2011	Imatinib Mesylate for the treatment of recurrent fibromatosis: Phase II, single arm	40	95% (2 year OS)	55% (2 year PFS)	11% 3 month RR [CR 1, PR 3, SD 28, PD 3]	45% (rash, abdominal pain, vomiting, nausea, diarrhea, myalgia, asthenia)
	Sunitinib (Jo et al.) [38]	2014	Sunitinib: Prospective multicenter phase II study	19	94.4% (2 year OS)	74.7% (2 year PFS)	26.3% ORR	Neutropenia (33.3%), diarrhea (5.3%), hand–foot syndrome (5.3%)
	Imatinib Mesylate (Kasper et al.) [39]	2017	Imatinib for RECIST progressive desmoid tumors: Phase II study of the German Interdisciplinary Sarcoma Group (GISG)	38	100% (2 year observational period)	45% (2 year PFS)	ORR 19%	Grade 4 neutropenia (2%), grade 3 toxicities (11%) (including neutropenia, leukopenia, nausea/vomiting, gastritis, rash, contracture)
	Sorafenib Trial (Gounder et al.) [34]	2018	Sorafenib for advanced and refractory desmoid tumors: Phase III, double-blind, randomized, placebo-controlled trial	87	NR	81% vs. 36% (2 year PFS)	33% vs. 20%	47% vs. 25%
	DESMOPAZ (Toulmonde et al.) [40]	2019	Pazopanib (P) or methotrexate-vinblastine (M/V): a non-comparative, randomized, open-label, multicenter, phase 2 study	72	NR	P: 85.6% vs. M/V: 79% (1 year PFS) P: 67.2% vs. M/V: 79% (2 year PFS)	NR	P: HTN (*n* = 10, 21%), diarrhea (*n* = 7, 15%); M/V: neutropenia (*n* = 10, 45%), liver transaminitis (*n* = 4, 18%)
γ-secretase inhibitors	DeFi Trial (Gounder et al.) [41]	2023	Nirogacestat versus placebo in adult patients: randomized, double-blind, placebo-controlled, phase III trial	142	NR	76% vs. 44% [2 year PFS]; HR = 0.29	41% vs. 8%	55% vs. 17%
	RINGSIDE Trial- AL102 (Kasper et al.) [42]	Ongoing	AL102 (Varegacestat) γ-secretase inhibitor for adults with progressing desmoid tumors: Phase II/III trial	198	NR	NR	ORR 64% (in prelim, phase II data)	Grade 3 toxicities 33%; Grade 4/5 toxicities 0% (in prelim, phase II data)

Patient (Pt), Not Reported/Not Reached (NR), Response Rate (RR), Complete Response (CR), Partial Response (PR), Stable Disease (SD), Progressive Disease (PD). For Phase III Trials, placebo is listed second where rates of response, toxicity, etc., are compared. Sarcoma Alliance For Research Through Collaboration (SARC). Response Evaluation Criteria In Solid Tumors (RECIST).

## Data Availability

No new data were created or analyzed in this study. Data sharing is not applicable to this article.
